# Sleep Quality Evaluation Based on Single-Lead Wearable Cardiac Cycle Acquisition Device

**DOI:** 10.3390/s23010328

**Published:** 2022-12-28

**Authors:** Yang Li, Jianqing Li, Chang Yan, Kejun Dong, Zhiyu Kang, Hongxing Zhang, Chengyu Liu

**Affiliations:** 1 School of Instrument Science and Engineering, Southeast University, Nanjing 210096, China; 2 School of Information Science and Engineering, Southeast University, Nanjing 210096, China; 3 Aerospace System Engineering Shanghai, Shanghai 201109, China; 4 State Key Laboratory of Proteomics, Beijing Proteome Research Center, National Center for Protein Sciences, Beijing Institute of Lifeomics, Beijing 102206, China

**Keywords:** sleep quality evaluation, single-lead ECG, sleep staging, heart rate variability, sleep

## Abstract

In clinical conditions, polysomnography (PSG) is regarded as the “golden standard” for detecting sleep disease and offering a reference of objective sleep quality. For healthy adults, scores from sleep questionnaires are more reliable than other methods in obtaining knowledge of subjective sleep quality. In practice, the need to simplify PSG to obtain subjective sleep quality by recording a few channels of physiological signals such as single-lead electrocardiogram (ECG) or photoplethysmography (PPG) signal is still very urgent. This study provided a two-step method to differentiate sleep quality into “good sleep” and “poor sleep” based on the single-lead wearable cardiac cycle data, with the comparison of the subjective sleep questionnaire score. First, heart rate variability (HRV) features and ECG-derived respiration features were extracted to construct a sleep staging model (wakefulness (W), rapid eye movement (REM), light sleep (N1&N2) and deep sleep (N3)) using the multi-classifier fusion method. Then, features extracted from the sleep staging results were used to construct a sleep quality evaluation model, i.e., classifying the sleep quality as good and poor. The accuracy of the sleep staging model, tested on the international public database, was 0.661 and 0.659 in Cardiology Challenge 2018 training database and Sleep Heart Health Study Visit 1 database, respectively. The accuracy of the sleep quality evaluation model was 0.786 for our recording subjects, with an average F_1_-score of 0.771. The proposed sleep staging model and sleep quality evaluation model only requires one channel of wearable cardiac cycle signal. It is very easy to transplant to portable devices, which facilitates daily sleep health monitoring.

## 1. Introduction

Sleep is a vital activity of humans. However, it is difficult to measure accurately. In clinical conditions, polysomnography (PSG) and sleep questionnaires are usually used as objective and subjective standards, respectively, for the assessment of sleep quality. PSG is useful in the diagnosis and treatment of sleep disorders [1]. With it, sleep stage transitions and automatic nervous system activities in sleep can be observed by multichannel physiological electric signals. The up-to-date interpretation of PSG by the American Academy of Sleep Medicine (AASM) divides all sleep time into five stages: stage of wakefulness, stages 1–3 of non-rapid eye movement (stage N1-N3 in NREM) and a stage of rapid eye movement (REM) [1,2]. This categorization helps the diagnoses of some sleep diseases; however, the PSG’s defects of inconvenience in daily use and interference with sleep are obvious and difficult to resolve. The sleep questionnaire is a subjective and rough assessment of the subject’s overall sleep quality. Both PSG and sleep questionnaires are essential tools to obtain an individual’s sleep health information; however, the connections between them are not so significant or coincident and cannot be indicated by simple mathematical expressions. Researchers have found that in healthy adults, subjective total sleep time is overrated compared with objective total sleep time, as well as subjective sleep efficiency [3]. Sleep stage N2 is closely correlated with subjective sleep quality and neither sleep efficiency nor total sleep time can be a predictor of subjective sleep quality. However, in older people, objective sleep efficiency is the strongest correlate of subjective sleep quality, but is inconsistent with that of younger adults. The restoration from the sleep of younger adults relates to slow-wave sleep closely [3,4].

Standard PSG monitoring obtains multiple signals such as electroencephalogram (EEG), electrooculogram (EOG), electrocardiogram (ECG), mouth and nose breathing and chest breathing. Among them, EEG is the most highly correlated with sleep staging results. Because PSG monitoring is complicated work with many wires, usually at least 12 leads, it interferes with patients’ normal sleep, so technical needs exist to simplify the PSG method without too much loss of accuracy in sleep staging. In the research field, by extracting one or several channels of PSG signals, sleep staging algorithms are commonly based on EEG [5], ECG [6,7] and cardiopulmonary coupling (CPC) [8]. Despite the fact that the algorithms based on EEG generally perform much better than those based on ECG and CPC [6,9,10], the discomfort and noisy signal of EEG are its bottleneck. We need reduced connecting lines and fewer foreign body sensations to make sure that sleep is not disturbed by devices and that the data more closely resemble natural sleep.

Many research studies have verified the possibility of sleep staging by ECG signals [6,8,11,12]. ECG signals can be much more easily obtained than EEG signals. We only need to collect one lead of wearable ECG signals and that is very convenient and offers small disturbance to subjects. The signals can be obtained by the portable devices with two or three electrodes pasted on the surface of the subjects’ chest or abdomen, with no wires around the body. Heart rate variability (HRV) is the physiological phenomenon of variation in the time interval between heartbeats. It is measured by the variation in the beat-to-beat interval. The data source used to calculate HRV is usually ECG or photoplethysmography (PPG); the difference between them is negligible [13,14]. Many researchers have introduced large numbers of HRV features and complicated neural networks into their sleep staging models, which is very time consuming and short of practical value [6,15,16,17,18], and this need to be altered and improved.

In practice, the demand exists to evaluate subjective sleep quality with a few physiological signals for ordinary persons and for special post staff. The special post staff are those whose working hours are lengthy, working content is difficult and the accommodation is rough, such as soldiers, firefighters, pilots and construction workers. They must be energized before performing a special task, which requires them to have a good sleep. Thus, it is better for their sleep quality to be supervised objectively by the leaders. However, it is not a wise option to have them fill out the sleep quality questionnaires because some unaware or intended false answers might be included. We should resort to some technical means to obtain sleep information objectively without significantly affecting the staff’s normal sleep. Generally, for healthy people, how they really feel about their sleep is considered to be credible and acceptable. The score of their subjective sleep questionnaires can be thought to be the standard of sleep quality. The aim of this paper is to develop a practical method to differentiate subjective sleep quality into two groups—“good” and “poor” using single-lead wearable cardiac cycle signals. To achieve this, we collected ECG data and sleep questionnaires from 200 employees who work in special positions when they sleep at night. We should first build a sleep staging model as the bridge. We strive to screen out those whose sleep quality is poor and demand that the algorithm does not consume too many hardware resources and can be easily transplanted into portable devices.

## 2. Materials

### 2.1. International Public Dataset

Our sleep staging model is built mainly based on the international public database and the following two datasets were used: (1) the Sleep Heart Health Study Visit 1 database (SHHS1DB) [19,20], (2) the PhysioNet Computing in Cardiology Challenge 2018 training database (CincDB) [15,21]. The SHHS1DB includes the PSG data of 6441 individuals; the participants have no history of treatment of sleep apnea, no tracheostomy, and no current home oxygen therapy. The CincDB is from a challenge whose goal is to classify target arousal regions in the sleep time at night using PSG data. In the training set of CincDB, 994 subjects were included, some of whom were diagnosed with sleep disorders. All the data we used are annotated in 30 s contiguous intervals: wakefulness, stage N1, stage N2, stage N3 and REM. We pick out only one channel of ECG signals from PSG data. We selected all the subjects in CincDB and the same number of subjects in SHHS1DB randomly.

### 2.2. Subjects

Two hundred healthy male subjects participated in this experiment. Their age ranged from 18 to 35. When they slept habitually in their bunks at night, each person was asked to paste a tiny device manufactured in our laboratory on their chest to record ECG signals. The size of the device is 87 × 54 × 16 mm, small enough that it cannot interfere with subjects’ normal sleep. There are two electrodes on the back side of the device. The sample frequency is 250 Hz and it can work continuously for 24 h. The storage is enough for 20 days’ record. The experiment lasted for one night. All the staff were required to fill in the sleep questionnaire the next morning as soon as they woke up.

The subjective sleep questionnaire was modified from the widely used Pittsburgh sleep quality index (PSQI) questionnaire [16]. A few questions relevant to the professional characteristics of the special position staff were added to the questionnaire and the questions related to general conditions of the past month’s sleep quality were replaced by the questions reflecting one night’s sleep conditions or deleted. The total score of our questionnaire ranges from 0 to 22 and the lower the score, the better the sleep experience. There are 7 components in the scoring system, 6 components ranging from 0–3, and 1 component ranging from 0–4. The questionnaire and the scoring system of it are contained in Appendix A. It should be considered that the special position staff are young people, most of them sleep well in normal conditions and they never use medicines to help them sleep. After being assessed by experts, the score below or equal to 6 indicates “Good” and above 6 indicates “Poor” in the grading criterion. In our experiment, subjects were divided into 2 classes—“good sleep” and “poor sleep”.

The study was approved by the Ethics Committee of Zhongda Hospital, affiliated to Southeast University, China. All the subjects were well informed of the aims and risks of the experiment and they all signed informed consent before the study.

## 3. Method

Sleep staging model building is the first step to implement sleep quality evaluation. The second step is to construct a sleep quality evaluation model as a bridge of objective sleep staging features and subjective sleep questionnaire scores.

### 3.1. Sleep Staging Model

The sleep staging model was built based on the international public dataset. Before model building, we divided the dataset into two parts: half of each database was the training set and the other half was the test set. For obvious absent data (data less than 5 h in length), data error, those with obvious sleep disease (whose Apnea–Hypopnea Index (AHI) is equal or more than 5), objective sleep efficiency less than 75%, and if the recording time began too early (over 45 min at the beginning is wake stage) or ending too late (over 30 min at the end of the data is wake stage), the subjects’ data were discarded.

#### 3.1.1. Data Preprocessing

The R waves of ECG signals were automatically identified by the P&T method for its extensively tested accuracy and efficiency [22]. According to the calculation rules of HRV parameters, abnormal heartbeats, such as wide gap longer than 2 s, narrow gap shorter than 0.35 s and irregular adjacent gaps, were removed automatically. If the wake time at the beginning of each subject’s signal is longer than 5 min, the extra signal before the last 5 min in the wake time is cut off. The sleep stages were annotated every 30 s. For each 30-s data segment, the 4 min 30 s data centered on it is the basic unit for calculating HRV features; the segments with more than two kinds of sleep stages were not included.

#### 3.1.2. Feature Selection

1. Candidate features that represent the characteristics of the data segment itself

The sleep staging model is based on the transformation of different HRV features in the whole night. Each HRV feature is calculated every 4.5 min. Thus, to build the model we should first select proper HRV features and calculate their values. In our work, some frequently used features in research and clinical conditions and two-time irreversibility features were taken into consideration in the feature selection procedure. We examined how each feature and each combination of features correlate with the transformation of sleep stages by analysis of variance. The 23 features we picked out are listed in Table 1. HR, SDNN, RMSSD, SDSD, PNN50, LF, HF, VLF, LF/HF ratio, LFn and HFn are linear HRV features. Among them, feature coRR, resf and strf are cited from Heenam Yoon’s work [23] and they are effective in the recognition of the REM stage. Feature ɑ1 and ɑ2 are the slope and offset of Detrended Fluctuation Analysis (DFA) respectively. The DFA method was first proposed by Peng et al. in 1994 when they studied the structure of DNA molecular chain [24]. DFA analysis first removes trends caused by external factors in the sequence, and then studies the long-range correlation of the sequence. DFA analysis can eliminate trend components of all orders, deal with those time series with long-range correlation and non-stationary data, and eliminate the pseudo-correlation phenomenon. *P*_1_ and *G*_1_ are time irreversibility parameters that measure time imbalance of a RR intervals sequence [25,26,27,28]. The difference value between adjacent RR intervals could be positive, negative and zero. The positive and negative ones are expressed by $\Delta RR^{+}$, $\Delta RR^{-}$ and their number are shown as $N\left(\Delta RR^{+}\right)$,$N\left(\Delta RR^{-}\right)$. The two features are defined in Formulas (1) and (2).

(1) $$P_{1}=\frac{N\left(\Delta RR^{-}\right)}{N\left(\Delta RR^{-}\right)+N\left(\Delta RR^{+}\right)}\times 100$$

(2) $$G_{1}=\frac{\sum_{i=1}^{N\left(\Delta RR^{+}\right)}\Delta RR^{+}{\left(i\right)}^{2}}{\sum_{i=1}^{N\left(\Delta RR^{+}\right)}\Delta RR^{+}{\left(i\right)}^{2}+\sum_{i=1}^{N\left(\Delta RR^{-}\right)}\Delta RR^{-}{\left(i\right)}^{2}}\times 100$$

If the length of a sequence is n, and the first and last n-2 points constitute sequences s_1_, s_2_ respectively, corr2 is the coefficient of s_1_ and s_2_.

**Figure 1:**
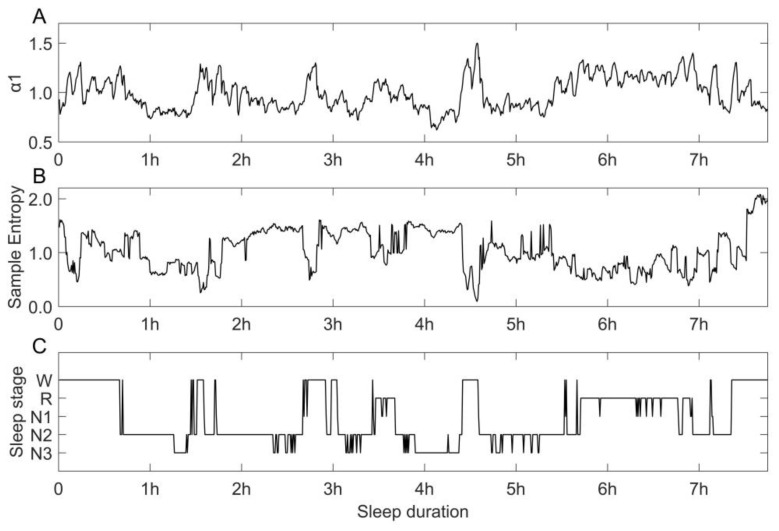
Variations in HRV parameter ɑ1, sample entropy and sleep stages in a whole night of a subject in SHHS1DB. (**A**–**C**) show the corresponding graph of ɑ1, sample entropy and sleep stages.

HRV parameters vary with changes of sleep duration and sleep stages. If we plot the variation trend of a HRV parameter in a sliding window whose length is five minutes and step size is 30 s in a whole night’s sleep, we can find out the regularities in how HRV parameters vary synchronously with the changing of sleep stages. Figure 1 is an example, demonstrating the synchronous relationship of the index ɑ1 and SE with sleep stages.

2 Candidate features that represent the relationship between current data unit and the adjacent/overnight data units

Considering that the feature value saltus is often accompanied by the transformation of sleep stages, the relationship between each data unit to be classified and the adjacent or overnight data units has a great influence on the classification results. In the process of classifier construction, we need to introduce new features to reflect whether there is a trend rise or fall in HR and respiration rate between two adjacent data units, and compare the level of HR and respiration rate of this data unit with that within one hour and overnight. Based on this, the features we extracted are listed in Table 2. The rising and falling trend features are the slope value between the first and second maximum points (t1, t2) and the first and second minimum points (b1, b2) are given in 3 min windows before and after current data unit.

3 Useless features elimination

In the process of model building, some features made little difference on the improvement of sleep stage recognition accuracy or could be replaced by other features. Thus, further feature selection is needed. For the features in Table 1, we limited the number of features to 2 or 3 and exhausted all the feature combinations to examine the recognition effect of different sleep stages. Then, the feature combinations were ranked in the order of recognition accuracy and Youden’s index. Youden’s index is a method to evaluate the authenticity of the screening test [29]. The index is the average performance of sensitivity and specificity of a classification result. It gives equal weight to false positive and false negative values, so all tests with the same value of the index give the same proportion of total misclassified results.

(3) Youden’s index=sensitivity+specificity−1

Features frequently demonstrating relatively high accuracy and Youden’s index were reserved and others were excluded. For feature combinations, we first computed the first principal component with principal component analysis (PCA) and it was smoothed at a small and a large frame length. An offset value was added on the large frame length smoothed curve. The detailed procedure is similar to building the recognizers of the model, which is introduced below in the section on “Steps of model construction”. When trying to recognize one sleep stage, some features pull down the average value of the combination’s performance and some have no remarkable effect. They are all useless for the recognition of a certain stage. Finally, we obtained four groups of “Useless features” and the features in their intersection are removed from the whole features set. This method reduces the number of features without losing the potential for the improvement of the model’s performance.

For the features in Table 2, Support Vector Machine methods based on Recursive Feature Elimination (SVM-RFE) was applied to implement the feature selection for each classifier. SVM-RFE method was first proposed by Guyon when he studied the gene selection for cancer classification [30]. This method sorts the features in the order of their significance based on the score of each feature, which helps us eliminate those features that make little difference to the performance of the model and reserve those that are useful to obtain the best accuracy.

#### 3.1.3. Steps of Model Construction

1. Principle of recognizers’ construction

**Figure 2:**
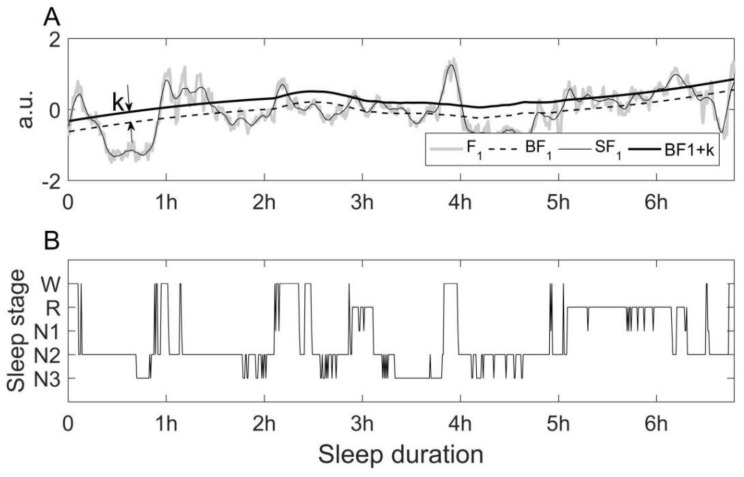
An example showing how to recognize wakefulness by the first principal component. (**A**) Variations of the first principal component for recognizing wakefulness segments. (**B**) Sleep stages. (F_1_ is the first principal component. SF_1_ and BF_1_ are the smoothed result of F_1_ at a small and a large frame length respectively. k is the translation range of BF_1_. The parts that meet SF_1_ > BF_1_ + k are the recognized wakefulness segments).

In our work, we first observe how the principal component of the selected features varies in the whole night relative to its smoothed tendency. We used Savitzky-Golay FIR filter as the smoothing filter. The relative position is an important basis for distinguishing different sleep stages. Our task is to look for feature combinations suitable for recognizing a specific sleep stage. To achieve this, we exhaust all the feature combinations, calculating their first principal component respectively, then transform them into their zero mean and unit variance. For a subject’s whole-night data, the first principal component F_1_ is smoothed at a small and a large frame length, named SF_1_ and BF_1_. SF_1_ follows the variation in F_1_ more closely and BF_1_ reflects the major transformation of sleep cycles in the night. Figure 2A is an example. F_1_ is the first principal component of a combination of four HRV feature (coRR, HR, resf and ɑ2). The relative position of SF_1_ to BF_1_ relates to how deep the sleep is. When SF_1_ is above BF_1_ and they are far from each other, a jump often appears in sleep stages from NREM sleep to wakefulness or REM sleep. Thus, we can recognize a specific sleep stage by the relative position of SF_1_ to BF_1_. In order to make the recognition effect better, the BF_1_ curve should be moved up or down a little bit. We used k to denote a small distance. We put the combination of four features (coRR, HR, resf, ɑ2) and offset k together and called it the wakefulness recognizer.

We apply the same procedures to look for the appropriate features combination and k to construct the REM recognizer, light sleep (N1 and N2 included) recognizer and deep sleep (N3 stage) recognizer respectively. For the recognition of wakefulness and REM sleep, the criterion is SF_1_ > BF_1_ + k, and for the recognition of deep sleep, the criterion is SF_1_ < BF_1_ + k. Furthermore, BF_1_ + k1 < SF_1_ < BF_1_ + k2 is used to recognize the light sleep stage. The criterion differs due to the characteristics of HRV features. When a subject is aroused from NREM sleep, a jump would occur from a relatively low value to a higher value in many major HRV features except SE and ɑ2, whose variation regularity is often inverse to other features. Before PCA, we should reverse the two features to their opposite number in order to achieve better accuracy.

2 Boundary value processing based on variable threshold and window width

**Figure 3:**
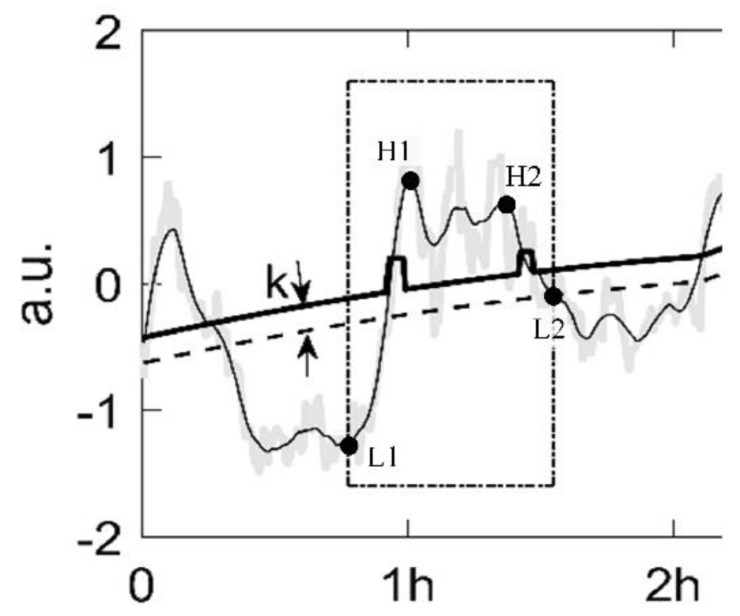
Graphical representation of variable threshold and window width (data are from Figure 2).

In the actual data analysis, if only BF_1_ ± k is used as the threshold to identify a certain sleep stage, the data segments at the intersection of SF_1_ and BF_1_ ± k are likely to be misjudged. We use variable windows and local threshold adjustment to correct the classification of W, R and D segments. First, the windows in which the height difference and slope between maximum points and minimum points meet the threshold requirements are found. Taking W recognizer as an example, the window starts from the minimum point of SF_1_ below BF_1_, along with the transformation of SF_1_ from negative to positive, passing the maximum point of SF_1_ above BF_1_ + k, until SF_1_ goes down through the intersection of BF_1_ (as shown in Figure 3). We performed threshold adjustment at the medial side of the intersections of SF_1_ and BF_1_ + k. The threshold of W and R recognizer is lifted, and D recognizer is in the opposite direction. The width and height of the threshold adjustment is proportional to the slope and height difference between the high point and low point on the same side (H1&L1, H2&L2).

3 Determination method of the final category

To classify ECG segments into four groups, a multi-classifier fusion method was applied. We first pass every ECG segment into the four recognizers to see which categories each ECG segment belongs to, respectively. A problem may occur if an ECG segment belongs to more than one sleep stage, so it is necessary to consider their grouping carefully. Here, the sigmoid-fitting method proposed by Platt [31] was used. In this method, the output result of the standard SVM is fitted with the LR model (sigmoid function), and the original output value of the model is mapped to the probability value [0, 1]. Firstly, we use SVM to build the WR/N classifier, W/R classifier and L/D classifier. Depending on the type and number of labels that may be attached to each data segment, they are selectively passed through each of the three classifiers. The data segments selected by multiple recognizers are first sent to the WR/N classifier. The probabilities they belong to W + R and N classes are P(WR) and P(N) respectively. Then they are passed through W/R classifier and L/D classifier to get the conditional probability P(W|WR), P(R|WR), P(L|N) and P(D|N). Therefore, the probability that each data segment belongs to the four categories is the joint probability of the above two categories. The classification to which the maximum probability belongs is taken as the final judgment result of the data segment, that is, the category corresponds to the maximum value among P(WR) × P(W|WR), P(WR) × P(R|WR), P(N) × P(L|N) and P(N) × P(D|N). For data segments whose labels only contain W & R, or L & D, we do not calculate the probability of P(WR) or P(N).

**Figure 4:**
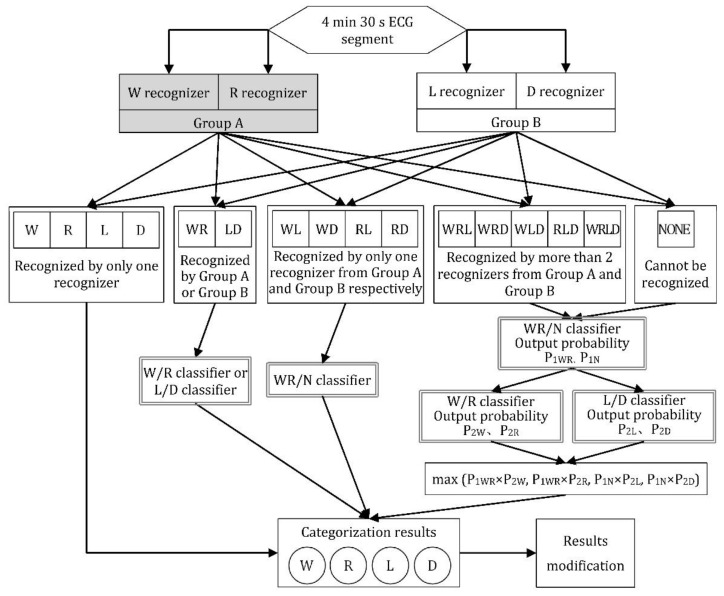
Graphical representation of classification procedure. (Classification procedure: Every ECG segment is detected by the four recognizers in turn. The sequence will be labeled by “W”, “R”, “L” or “D” if it passes the correspondent recognizer. The tags on each sequence are unequal in number. For example, “WR” indicates the sequence passes the W recognizer and R recognizer at the same time. For those segments with two tags, the WR/N classifier, W/R classifier and L/D classifier perform the judge function to decide which category they belong to. For those segments with 0 or more than 2 tags, we determine the final classification result by calculating the probability that they belong to each category.).

The procedure to operate this classification algorithm is demonstrated in Figure 4. Every 4.5 min ECG data segment is first recognized by the four recognizers (wakefulness recognizer, REM stage recognizer, light sleep recognizer and deep sleep recognizer) which are abbreviated to W recognizer, R recognizer, L recognizer and D recognizer, respectively. Each recognizer is responsible for determining whether this RR intervals sequence belongs to its category. If yes, we label this RR intervals sequence with the tag of the recognizer (W, R, L and D). After being checked by all four recognizers, each RR intervals sequence is attached with tags of different number, ranging from zero to four. Based on the category and number of the tags, different strategies are applied. (1) For the RR intervals sequence with only one tag, they are already categorized. (2) For the RR intervals sequence with two tags, if the two tags are from recognizer Group A (W and R recognizer) or Group B (L and D recognizer), that is, the tags on the sequence are WR or LD, they are divided further by classifiers used to differentiate W and R, or L and D. If the two tags are from Group A and Group B respectively (WL, WD, RL, RD), they are decided by WR/N classifier. (3) For the RR intervals sequence with no tag or with three or four tags, they are first sent to WR/N classifier to obtain the probability values P_1WR_ and P_1N_, and then sent to the W/R classifier and L/D classifier to obtain the conditional probabilities P_2W_, P_2R_, P_2L_ and P_2D_. The final classification result corresponds to the maximum joint probability of the above two steps’ probability.

After the classification procedure, the results could be modified to conform to the physiological laws of real sleep. The REM stages which emerge too early (in the first 80 min after sleep begins) should be replaced by wakefulness, and deep sleep in the half hour before waking up was modified to light sleep.

#### 3.1.4. Model Performance Guarantee and Examination

Ten-fold cross validation was used in the training set when we built the model. The test set was divided randomly into five equal parts which were named K1 to K5 for the examination of the results balance. We used the accuracy and average F_1_-score to evaluate the performance of the model. F_1_-score is the harmonic mean of the precision and recall. Precision is also known as positive predictive value, and recall is also known as sensitivity in binary classification.

### 3.2. Sleep Quality Evaluation Model

#### 3.2.1. Data Preprocessing

The data sources for this model are the subjects’ ECG data and their sleep questionnaires. The ECG data covering less than 5 h or with too much noise mixed in (noise occupying more than 25% of data) was discarded. If there is too much information omitted to calculate the total score, the questionnaire together with the ECG data will also be excluded. Finally, there were 113 subjects left. The data information is listed in Table 3. Half of them were categorized into the training set and the other half into the test set randomly. The subjects belonging to each group were distributed equally in the two sets.

#### 3.2.2. Model Principle

We aimed to divide all subjects into two groups, “good sleep” and “poor sleep”, according to objective sleep quality features. Subjective sleep score acts as a standard and verification.

From the sleep staging results, we mainly extracted features that can reflect sleep fragmentation, sleep depth and sleep duration. For the measurement of sleep fragmentation, we calculated the occurrence number of successive W stages and the intensity of sleep stage transitions. Here, the intensity of sleep stage transitions is defined as follows:

First, we labelled W, R, and L/D stages 1, 0 and 1 respectively. Then, we calculated the absolute value of the difference of the two neighboring labels. Finally, we summed up all the absolute differences as the intensity of sleep stage transitions.

Other features include sleep efficiency, sleep and REM incubation, total sleep and different sleep stages duration, proportion of different sleep stages, wakefulness times, and the frequency of W and R labels after 6 h since the beginning (measurement of early awakening).

The SVM-RFE method was also used to conduct feature selection. We also adopted accuracy and F_1_-score as the standard of the model’s performance and five-fold cross validation was used.

## 4. Results

### 4.1. Feature Selection Result

**Figure 5:**
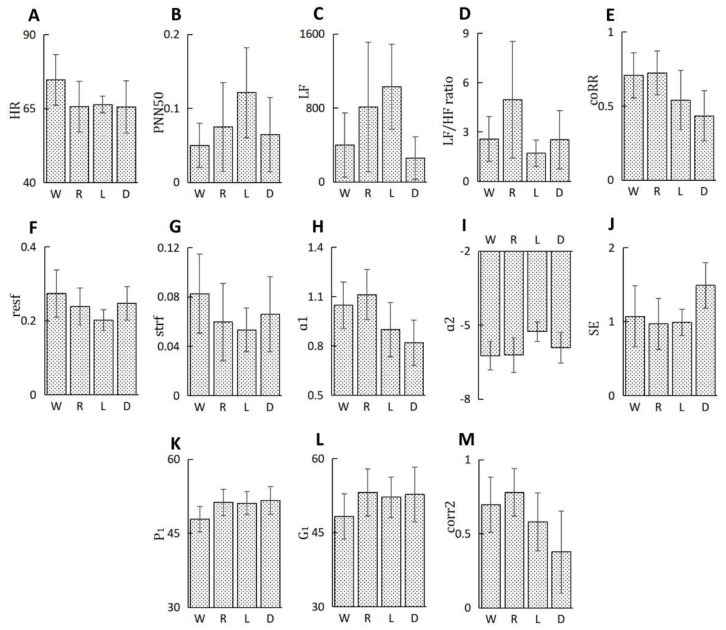
(**A**–**M**) show the statistical difference of the features we select in different sleep stages. (“W”,” R”,”L”,”D” are the abbreviation of wakefulness stage, REM stage, light sleep (N1&N2) and deep sleep (N3) respectively. Aesthetically, the vertical axis representing ɑ2 is plotted in reverse order).

From the features set, there are 13 HRV features left for the building of the recognizers and the features that represent the mutual relationship of data units are all essential in the construction of the classifiers. The 13 HRV features are HR, PNN50, LF, LF/HF ratio, coRR, resf, strf, ɑ1, ɑ2, SE, P_1_, G_1_ and corr2. Variance analysis was implemented for every feature to prove their distinguishing ability for at least one sleep stage. The result suggests that every feature demonstrates a high significant difference for at least one sleep stage. Statistical difference of all the features in different sleep stages is demonstrated in Figure 5 using the mean ± standard deviation format. Each subfigure represents a specific feature, and we can clearly see that their mean value varies with the sleep stages. For example, the feature SE in D stage is distinctly larger than the other three stages, and ɑ2 in L stage is much smaller than the other three stages. Successive two samples u test result shows the p values are all less than 0.001 which indicates that all features make a difference picking the four stages out from all ECG segments.

Experiment results showed that the combination of specific features which are suitable for the recognition of a certain sleep stage are not applicable for the recognition of other sleep stages. The combination of features that suit each recognizer best is listed in Table 4.

For the sleep quality evaluation model, nine features are left and they are sleep efficiency, total sleep time, the occurrence number of successive W stages, the intensity of sleep stage transitions, and the frequency of W and R labels after 6 h since the beginning, NREM and W duration, R and D proportion. The third-order polynomial kernel was the most fitting for the model by testing.

### 4.2. Performance of Sleep Staging Model

The algorithm has been tested in CincDB and SHHS1DB and the results of four classes’ categorization (wakefulness, REM, light sleep and deep sleep) are listed in Table 5. There is not huge difference in accuracy and F_1_-score between the two databases. Accuracy is the average value of all data and F_1_-score is the mean value of different categories. We obtained an accuracy of 0.661 and an F_1_-score of 0.625 in CincDB. The accuracy and F_1_-scores in SHHS1DB were 0.659 and 0.624 respectively. The confusion matrixes are in Appendix B (Table A1 and Table A2).

### 4.3. Performance of Sleep Quality Evaluation Model

The results of each fold are demonstrated in Table 6. The accuracy in the test set is 0.786 and the average F1-score is 0.771 at the same time.

## 5. Discussion

In this paper, we proposed a scheme that is easy to transplant to the common wearable ECG devices to conduct sleep quality evaluation and applied it to practical conditions. Our model has been tested by the international public database and medically approved sleep questionnaires involving over 100 subjects. In the model building procedure, a new method for sleep staging using single-lead wearable ECG signals has been developed and the mapping relationship between objective sleep parameters and subjective sleep questionnaires has been expressed by a SVM model.

In the sleep staging model building procedure, we balanced light weight and performance. We did not use too many features or a complex computing method. Researchers have pointed out the good coincidence of HR & pulse rate (PR), HRV & pulse rate variability (PRV) [13]; thus, we can also obtain pulse intervals as a replacement of cardiac intervals. The pulse acquisition devices are more easily accessible. Our scheme suggests a possible way of applying the cardiac cycle sequence to sleep quality. This is important for the routine testing of special post staff and ordinary persons.

We also tried our best to construct a model with a clear frame which provides a reasonable possibility for further modification. With the development of hardware power, complex machine learning and deep learning methods have become popular for their good performance. However, abstract neural networks are like a black box; it is unclear what is inside. This limits the promotion and revision of the model. Clarity and ease of understanding are the advantages of our model and this is conducive for model migration to populations of different ages or disease.

In the process of sleep stage model building, feature selection is a critical step. In the process of constructing recognizers and classifiers, further feature selection was implemented to achieve the best accuracy and Youden’s index. Table 7 is an example of feature selection for recognizing the REM stage. The features combinations of high and low accuracy and Youden’s index are listed and the features existing in the upper half of the table occupy Important positions in REM stage recognition; in contrast, the features appearing in the bottom half of the table do not play a key role. From Table 7 we can find that LF, HF, VLF, LFn, HFn, SD1, SD2, SDSD, SDNN, RMSSD pull down the accuracy and FE, SE have no remarkable effect, so they are useless to REM. This method helps reduce the feature quantity to 13.

In Table 4, we notice that the feature combination of each recognizer is quite different. BF_1_ is the large frame length smoothed filtering result reflecting the transformation cycle of the whole sleep. HRV features are measurement of sympathetic and parasympathetic nerve activities, which is quite different from EEG. Thus, the algorithm accuracy based on ECG signals performs more poorly than algorithms based on EEG signals, which are the most important part in PSG sleep staging. Table 8 lists the four classes’ grouping performance in other researchers’ work. Some used ECG signals only and some added respiratory inductance plethysmography signals to build the model. In addition, the data source, data processing and result evaluation methods they chose are not the same. It is hard to compare the performance of models only by ranking the accuracy strictly.

Youden’s index is a measurement of the balance and credibility of the experimental results. It is a combination of sensitivity and specificity. Sensitivity is the accuracy of the target group recognition and specificity is the ability to pick out non-target groups precisely. For every link in the process of model building, considerations of Youden’s index are as important as target group accuracy. Ten-fold cross validation was used to deal with the potential over-fitting problem, although the phenomenon of over-fitting was not apparent regardless of whether the volume of training set was small or big because of the visualization of our model’s design thinking and since every ECG segment is considered in the whole night sleep cycle rather than separately and unrelatedly tested by a machine learning model. The large frame length smoothed trend line BF_1_ behaves as the comparative standard of the principal component of the combination of HRV parameters to substitute for an invariable threshold value in the model’s design thinking. This is because the same value range may correspond to different sleep stages for a specific feature with sleep progressing. Despite all that, individual differences are inevitable and that is the main source of error and accuracy decline.

**Figure 6:**
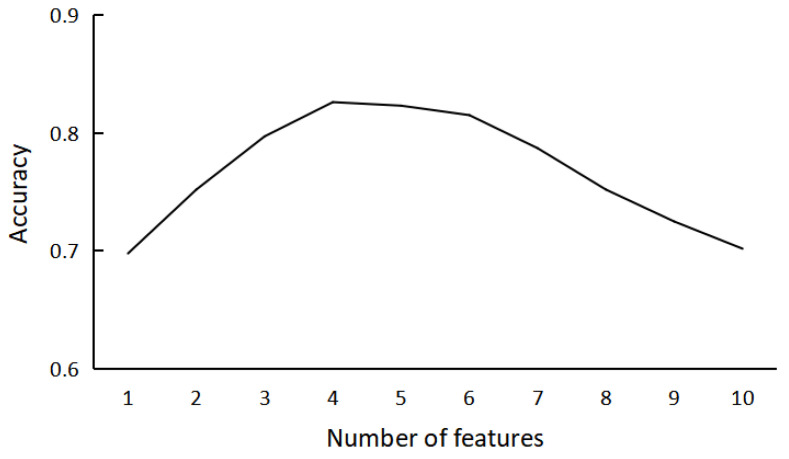
The relationship between the accuracy of W recognizer and its number of features. (The accuracy is the highest after comprehensive consideration of Youden’s index).

In addition, the performance of each recognizer is affected by the feature quantity, k value and the frame length of smoothing filter. We take W recognizer as an example to show how the accuracy correlates with feature quantity (Figure 6). The accuracy with a certain number of features is the highest with the best feature combination and with suitable Youden’s index.

**Figure 7:**
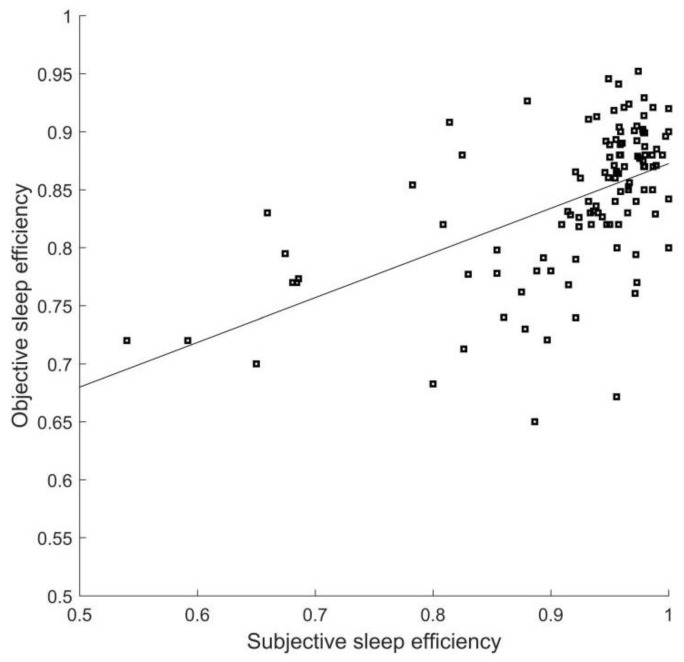
Corresponding relationship between subjective and objective sleep efficiency (Each spot represents a subject. The straight line is their least-square fitting curve).

**Figure 8:**
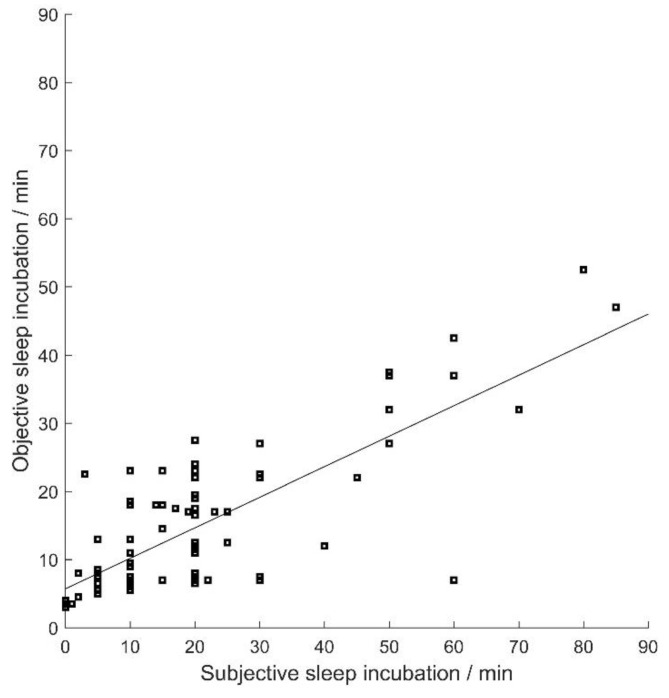
Corresponding relationship between subjective and objective sleep incubation (Each spot represents at least one subject for some of spots overlap. The straight line is their least-square fitting curve).

Differences exist between subjective and objective sleep parameters. Sleep efficiency is the ratio of the total time one is asleep to the total time one is dedicated to sleep. Sleep incubation is the time interval between one beginning to try sleeping and one falling asleep. We take them as examples. We calculate the objective and subjective sleep efficiency of every subject and plot a scatter diagram shown in Figure 7. From Figure 7 we notice that the sleep efficiency of the majority of subjects is greater than 0.8, and objective sleep efficiency tends to be smaller than subjective sleep efficiency. Similar results for sleep incubation are demonstrated in Figure 8. This is in accord with [5], although our sleep staging results are not the accurate PSG results.

With respect to the connection between subjective and objective sleep quality, there are studies revealing the correlation of subjective and objective sleep parameters with significance level p. However, only p is useless for the prediction work. No precise numerical connections between objective and subjective sleep quality parameters have been disclosed and we tried to understand the mapping relationship from sleep staging parameters to the questionnaire score using a machine learning method; however, apparent correspondence cannot be found, so we seek the second best outcome by just categorizing sleep quality into two classes and trying our best to meet practical demands.

There are still some limitations in this study, which should be addressed and improved in future studies. First, since most special position staff are currently male, we did not study gender differences in this work. This is a limitation of the work, and more female subjects need to be included in the experiment to explore the possible impact of gender differences to enhance the generalization of the model. Second, the dataset of this study excludes the data of most patients with sleep apnea. Sleep disorders and their sleep structures deserve more attention and further exploration. Night-to-night variability also needs to be considered in the model building. Third, since our research still needs to be improved on the precise discrimination of sleep stages by ECG, the sleep staging methods based on RR intervals can only be applied to daily home monitoring or rough assessment of sleep status of special positions staff, which is far from the requirements of clinical diagnosis. To bridge this gap, more basic studies on ECG are needed. Fourth, for practical applications, more real data for testing are needed. Fifth, cross-dataset validation is very important in improving the model’s generalization ability. Without it, when the model is applied to other datasets, the reliability of the prediction results will be affected. Therefore, improving the cross-dataset performance of the model is one of the key problems in the future. Sixth, since we have no access to accurate sleep stage labeling of special position staff, we cannot figure out the exact relationship between sleep stages and sleep quality. Despite PSG interfering with sleep, such data are important to improve the performance of the sleep quality evaluation model. Finally, the applicability and migration of the algorithm between different populations and different diseases need to be modified and improved in practice. Pre-study of each person is a good method to overcome individual differences.

## 6. Conclusions

This paper aims at developing a sleep quality evaluation strategy with single-lead wearable cardiac interval signals, so that the method can be easily transplanted into portable electronic products. To achieve this, we first use PCA, alterable boundary and multiple-classifier fusion method to build a sleep staging model. After that, we established a sleep quality evaluation model with the sleeping time ECG data and sleep questionnaires from 200 persons. The SVM and SVM-RFE methods were used. Although some limitations exist, our model reached a decent performance with fewer features and an explicable frame structure. This provides the possibility for daily home use or routine testing in working conditions if PSG is not easily accessible. The future work is to optimize and improve the algorithm to suit different populations, ages and sleep diseases.

## Figures and Tables

**Table 1:** Heart rate variability (HRV) candidate features that represent the characteristics of the data segment itself for sleep staging model.

Category	Feature	Description
Linear HRV features	HR	Average heart rate
	SDNN	The standard deviation of NN intervals.
	RMSSD	The root mean square of successive differences.
	SDSD	The standard deviation of the difference of the adjacent NN intervals.
	PNN50	The number of times in which the change in successive normal sinus intervals exceeds 50 ms.
	LF, HF VLF LF/HF ratio	Low-frequency and high-frequency power. Very low-frequency power. The ratio of low-frequency power to high-frequency power
	LFn, HFn	The normalized low-frequency and high-frequency power.
Features relevant to respiration	coRR	Coefficient of an RR interval sequence and its second-order smoothed sequence
	resf	The dominant frequency in the range of 0.15 to 0.5 Hz
	strf	The standard deviation of resf
Nonlinear features	ɑ1, ɑ2	The value of slope and offset in DFA.
	SE, FE	Sample entropy and Fuzzy entropy
	SD1, SD2	Poincaré plot features. SD1 and SD2 are the length of semi-minor axis and semi-major axis of the fitting ellipse of the Poincaré plot.
Time irreversibility features	*P*_1_, *G*_1_	As shown in formula (1) and (2)
Other features	corr2	If the length of a sequence is n, and the first and last n-2 points constitute sequences s_1_, s_2_ respectively, corr2 is the coefficient of s_1_ and s_2_.

**Table 2:** Candidate features that represent the relationship between current data unit and the adjacent/overnight data units.

Feature	Description
aHRs_t1_b1,aHRs_t1_b2, aHRs_t2_b1, aHRs_t2_b2, aress_t1_b1, aress_t1_b2, aress_t2_b1, aress_t2_b2, zHRs_t1_b1, zHRs_t1_b2, zHRs_t2_b1, zHRs_t2_b2, zress_t1_b1, zress_t1_b2, zress_t2_b1, zress_t2_b2	Rising and falling trend features of instantaneous HR and respiratory rate in the 3 min window before and after current data unit.
HRm_m1, resm_m1	The ratio of average HR and respiration rate in current data unit to that in current one-hour.
HRm_ma, resm_ma.	The ratio of average HR and respiration rate in current data unit to that in overnight data.

**Table 3:** Data information for sleep quality evaluation model. (Good and poor sleep are divided based on sleep questionnaires score).

Total Subjects	Deserted	Usable	
		Good Sleep (≤6 Points)	Poor Sleep (>6 Points)
200	87	74	39

**Table 4:** Feature combinations that suit each recognizer best.

Recognizer/Classifier	Features Combinations
W recognizer	coRR, HR, resf, ɑ2
R recognizer	coRR, HR, resf, ɑ1, ɑ2, G_1_
L recognizer	HR, resf, SE, ɑ2, P_1_
D recognizer	resf, LF, ɑ1, corr2

**Table 5:** Performance of sleep staging model.

Categories	Database	Performance in Each Subset			Accuracy	Average F_1_-Score
		Subset	Accuracy	Average F_1_-Score		
W/REM/N1, N2/N3	CincDB	K1	0.658	0.622	0.661	0.625
		K2	0.659	0.621		
		K3	0.665	0.628		
		K4	0.659	0.623		
		K5	0.663	0.629		
	SHHS1DB	K1	0.664	0.618	0.659	0.624
		K2	0.653	0.622		
		K3	0.656	0.623		
		K4	0.651	0.629		
		K5	0.670	0.628		

**Table 6:** The performance of sleep quality evaluation model.

“Good Sleep” Accuracy	“Poor Sleep” Accuracy	Average Accuracy	Average F_1_-Score
0.784	0.789	0.786	0.771

**Table 7:** Method of excluding “useless features” for REM stage recognition.

Features Combination	Accuracy/ Youden’s Index	Features Combination	Accuracy/ Youden’s Index
coRR, G_1_	0.799/0.538	coRR, resf, ɑ2	0.807/0.560
coRR, ɑ2	0.796/0.523	coRR, strf, ɑ2	0.805/0.557
ɑ1, ɑ2	0.789/0.530	ɑ2, P_1_, corr2	0.799/0.554
ɑ2, corr2	0.780/0.539	HR, ɑ2, corr2	0.795/0.548
ɑ1, P_1_	0.776/0.516	HR, ɑ1, ɑ2	0.794/0.536
corr2, P_1_	0.775/0.481	coRR, HR, ɑ1	0.789/0.531
coRR, ɑ1	0.769/0.480	LF/HF ratio, ɑ1, ɑ2	0.772/0.532
coRR, HR	0.758/0.481	coRR, PNN50, corr2	0.770/0.521
…	…	…	…
LF(HF), ɑ2	0.648(0.629)/0.315(0.298)	ɑ1, ɑ2, SD1(SD2)	0.721(0.718)/0.415(0.409)
VLF, ɑ1	0.609/0.226	ɑ1, ɑ2, SDNN(RMSSD)	0.712(0.717)/0.382(0.391)
LFn(HFn), ɑ1	0.596(0.607)/0.209(0.214)	coRR, strf, SDSD	0.704/0.411

**Table 8:** Comparison with similar works (four classes).

Work	Signals	Database	Feature Quantity	Method	Accuracy
Li Q. [32]	ECG	CincDB, SHHS1DB	8	CNN + SVM	0.656 0.659
Fonseca P [11]	ECG + RIP	48 of SIESTA	142	multi-class Bayesian linear discriminant	0.69
Sani M. Isa [33]	ECG	SLPDB	9	KDR + kNN + RF + SVM	0.60
Our work	ECG	CincDB, SHHS1DB	33	PCA + SVM	0.661 0.659

SLPDB: MIT-BIH Polysomnographic Database; SIESTA: a polygraphic and clinical database [34]; RIP: respiratory inductance plethysmography; CNN: Convolutional Neural Networks; RF: random forest; KDR: kernel dimensionality reduction.

## Data Availability

Not applicable.

## Appendix A

**Sleep Log Questionnaire**
**Job NO. Sex Age Date Device NO.**________ **Instructions:** *The following questions relate to your sleep quality last night. Please fill in the questionnaire as soon as you wake up in the morning. Your answers should indicate the most accurate reply of your real sleep quality.*

**1. Sleep time**
**a.** Time you went to bed last night:
**b.** Time you were ready to sleep last night:
**c.** Time you fell asleep last night:
**d.** Time you wake up in the morning:
**e.** Duration you were awake in the sleep:
**2. For each of the remaining questions, check the one best response. Please answer all questions.**

**a.** Wake up in the middle of the night or early morning	①Not	②Once	③Twice	④Three or more times
**b**. Have to get up to use the bathroom	①Not	②Once	③Twice	④Three or more times
**c.** Cannot breathe comfortably	①Not	②Once	③Twice	④Three or more times
**d**. Cough or snore loudly	①Not	②Once	③Twice	④Three or more times
**e.** Feel too cold	①Not	②Once	③Twice	④Three or more times
**f.** Feel too hot	①Not	②Once	③Twice	④Three or more times
**g.** Had bad dreams	①Not	②Once	③Twice	④Three or more times
**h.** Have pain	①Not	②Once	③Twice	④Three or more times
**i.** Wake up by noise or roommate	①Not	②Once	③Twice	④Three or more times
**j.** Wake up by physical cause or sleeping posture	①Not	②Once	③Twice	④Three or more times
**k.** Wake up by other reason(s)	①Not	②Once	③Twice	④Three or more times

**3. Feelings in the morning**

**a.** Feel energetic?	①Very energetic	②Fairly energetic	③Fairly sleepy	④Very sleepy
**b.** Head clear or dazed	①Very clear	②Fairly clear	③Fairly dazed	④Very dazed
**c.** How would you rate your sleep quality?	①Very good	②Fairly good	③Fairly bad	④Very bad

**4. How often have you taken medicine (prescribed or “over the counter”) to help you sleep?**

①Not during the past month	②Less than once a week
③Once or twice a week	④Three or more times a week

**Scoring system**

The items in Sleep Log Questionnaire are combined to form 7 “component” scores, each of which has a range of O-3 or 0–4 points. In all cases, a score of “0” indicates no difficulty, while a score of “3” or “4” indicates severe difficulty. The 7 component scores are then added to yield one “global” score, with a range of O-22 points, “0” indicating no difficulty and “22” indicating severe difficulties in all areas.

**Component 1: Subjective sleep quality**
Examine question 3c, and assign scores “0”, ”1”, ”2”, ”3” to the options “①”, “②”, “③”, “④” respectively.
**Component 2: Sleep latency**
Examine questions 1b and 1c, and calculate their time difference. Assign scores as follows:

Time difference between 1b and 1c	≤15 min	16~30 min	31~60 min	≥ 60 min
Score	0	1	2	3

**Component 3: Sleep duration**
Examine questions 1d and 1c, and calculate their time difference. Assign scores as follows:

Time difference between 1d and 1c	>7 h	6~7 h	5~6 h	<5 h
Score	0	1	2	3

**Component 4: Sleep efficiency**
Examine questions 1d, 1b and 1c, sleep efficiency is calculated as (1d-1c)/(1d-1b). Assign scores as follows:

Sleep efficiency	≥85%	75% ~84%	65% ~74%	≤ 65%
Score	0	1	2	3

**Component 5: Sleep disturbances**
Examine questions 2a-2k, and assign scores “0”, ”1”, ”2”, ”3” to the options “①”, “②”, “③”, “④” respectively. Add the scores for questions 2a-2k and assign scores as follows:

Sum of 2a-2k	0	1–9	10–18	19–27	27–33
Score	0	1	2	3	4

**Component 6: Daytime dysfunction**
Examine questions 3a-3b, and assign scores “0”, ”1”, ”2”, ”3” to the options “①”, “②”, “③”, “④” respectively. Add the scores for questions 3a-3b and assign scores as follows:

Sum of 3a-3b	0	1–2	3–4	5–6
Score	0	1	2	3

**Component 7: Use of sleeping medication**
Examine question 4 and assign scores “0”, ”1”, ”2”, ”3” to the options “①”, “②”, “③”, “④” respectively.
Global sleep score is the sum of the 7 components score.

## Appendix B

**Table A1:** Confusion matrix of the classification result in the test set of CincDB.

Classified as ↓	W	R	L	D
W	6388	1795	3970	418
R	1930	5601	5050	277
L	710	1196	23,060	902
D	117	63	4589	6013

**Table A2:** Confusion matrix of the classification result in the test set of SHHS1DB.

Classified as ↓	W	R	L	D
W	8325	2147	5198	395
R	2388	7210	6092	310
L	985	1334	27,869	1021
D	320	173	5412	6497
